# Effects of the SARS-CoV‑2 pandemic on residency training in orthopedics and traumatology in Germany: A nationwide survey

**DOI:** 10.1007/s00132-022-04295-w

**Published:** 2022-08-25

**Authors:** Dominik Adl Amini, Marit Herbolzheimer, Patricia Maria Lutz, Lucca Lacheta, Lisa Oezel, Henryk Haffer, Friederike Schömig, Anna Schreiner, Jonas Limmer, Maximilian Muellner

**Affiliations:** 1 Center for Musculoskeletal Surgery, Charité – Universitätsmedizin Berlin, corporate member of Freie Universität Berlin, Humboldt-Universität zu Berlin and Berlin Institute of Health, Charitéplatz 1, 10117 Berlin, Germany; 2 Department for Trauma Surgery, BG Trauma Center Murnau, Murnau, Germany; 3 Department for Trauma Surgery, Feldkirch Academic Hospital, Feldkirch, Austria; 4 Department of Orthopedic Surgery and Traumatology, University Hospital Duesseldorf, Duesseldorf, Germany; 5 Department for Trauma and Reconstructive Surgery, BG Unfallklinik Tübingen, University of Tübingen, Tübingen, Germany; 6 Department of Trauma, Hand, Plastic and Reconstructive Surgery, University Hospital of Würzburg, Würzburg, Germany

**Keywords:** COVID-19, Orthopedic education, Residency, Germany, Trauma surgery, COVID-19, Facharztweiterbildung, Deutschland, Orthopädie, Unfallchirurgie

## Abstract

**Background:**

The spread of the coronavirus disease has impacted healthcare systems worldwide; however, restrictions due to the SARS-CoV‑2 (severe acute respiratory syndrome coronavirus 2) pandemic are particularly drastic for physicians in residency training. Imposed restrictions interrupt the standard educational curricula, and consequently limited residents to meet mandatory requirements.

**Aim:**

The aim of this study was to evaluate the effects of the SARS-CoV‑2 pandemic on residency training in orthopedics and trauma surgery in Germany.

**Methodology:**

An online-based, voluntary, and anonymous survey of physicians in residency training for orthopedics and trauma surgery was conducted. Through email lists of junior physician organizations the survey was sent to 789 physicians. Participation was possible between October and November 2021.

**Results:**

A total of 95 participants (female 41.1%) with a mean age of 31.3 ± 2.8 years were analyzed. In the everyday clinical practice and care 80% of participants thought that they were set back in time of their general training due to the pandemic. There was an average reduction of 25.0% in time spent in the OR and 88.4% agreed that their surgical training was delayed due to the pandemic. Of the respondents 33.6% were able to attend external continuing education courses. Only 4.2% were able to invest more time in research and 55.8% of participants agreed that their residency training will be extended due to the pandemic.

**Conclusion:**

The COVID pandemic has had a significant impact on the residency training in orthopedics and trauma surgery in Germany. In almost all areas of training, residents had to accept restrictions due to the imposed restrictions, which potentially negatively affected their training.

**Supplementary Information:**

The online version of this article (10.1007/s00132-022-04295-w) contains the german and english version of the survey.

## Abstract (de)

**Hintergrund:**

Die Ausbreitung des Coronavirus hat die Gesundheitssysteme weltweit beeinflusst. Restriktionen aufgrund der SARS-CoV‑2(severe acute respiratory syndrome coronavirus 2)-Pandemie sind jedoch besonders einschneidend für ÄrztInnen in Weiterbildung. Auferlegte Restriktionen unterbrechen die vorgesehenen Ausbildungsetappen und führen zu möglicher Nichteinhaltung von obligaten Anforderungen.

**Ziel:**

Ziel der Studie war es, die Effekte der SARS-CoV‑2-Pandemie auf die Facharztweiterbildung im Fachbereich Orthopädie und Unfallchirurgie (O&U) in Deutschland auszuwerten.

**Methodik:**

Eine online-basierte, freiwillige und anonyme Befragung von ÄrztInnen in Weiterbildung im Fachbereich O&U wurde durchgeführt. Mittels E‑Mail-Verteiler von ärztlichen Nachwuchsorganisationen (Junges Forum O und U, Forum AGA Assistenzärzte) wurde die Umfrage an 789 ÄrztInnen gesendet. Die Teilnahme war zwischen Oktober und November 2021 möglich.

**Ergebnisse:**

Es wurden 95 Teilnehmer (w:41,1 %) mit einem Durchschnittsalter von 31,3 ± 2,8 Jahren analysiert. Im Klinikalltag denken 80 %, dass sie durch die Einschränkungen zeitlich zurückgeworfen wurden. Die Zeit der aktiven Teilnahme im OP reduzierte sich im Durchschnitt um 25,0 % und 88,4 % stimmten zu, dass sich ihre operative Ausbildung pandemiebedingt verzögern wird. Die Teilnahme an externen Fortbildungskursen war bei 33,6 % möglich. Nur 4,2 % konnten mehr Zeit in Forschung investieren. Insgesamt sind 55,8 % der Meinung, dass sich die Zeit der Facharztweiterbildung pandemiebedingt verlängert.

**Schlussfolgerung:**

Die COVID-19-Pandemie hat einen wesentlichen Einfluss auf die Facharztweiterbildung im Fachbereich O&U in Deutschland. In fast allen Bereichen der Weiterbildung mussten AssistenzärztInnen aufgrund der auferlegten Restriktionen Einschränkungen hinnehmen, die ihre Weiterbildung potenziell negativ beeinflussten.

## Introduction

In December 2019, the novel coronavirus disease 2019 (COVID-19) caused by the severe acute respiratory syndrome coronavirus 2 (SARS-CoV‑2) was first reported in Wuhan, located in the province of Hubei of the People’s Republic of China [10, 21, 22, 28]. Since then, the virus rapidly spread throughout the world reaching Germany on 27 January 2020 and was declared as a pandemic by the World Health Organization (WHO) on March 11th, 2020 [1, 3, 21, 26]. Furthermore, in March 2020, the German government decided on implementing the first lockdown and started reconstructing the healthcare system in order to maximize hospital capacity for the treatment of COVID-19 patients [3]. After gradually easing restrictions from June 2020 on, with the emergence of a second, third and fourth COVID-19 wave, the government was confronted with the need to reintroduce these restrictions and they were subsequently aggravated which once more affected the healthcare system [3, 11].

With these policies in place, COVID-19 has significantly impacted the healthcare system in general and surgical departments in particular for nearly 2 years [10, 18, 20, 23, 25]. To ensure adequate hospital recourses for COVID-19 patients, non-emergency surgical procedures had to be postponed or cancelled, and personnel was shifted to intensive care units and emergency departments intermittently [2, 25]. Furthermore, outpatient clinics scaled back drastically, cancelled all non-essential face-to-face appointments and implemented remote telemedicine appointments [8, 12]. Moreover, in university hospitals, lectures and educational formats were cancelled in order to uphold the governmentsʼ mandated contact restrictions [8].

All of these restrictions are crucial for resident physicians in orthopedics and traumatology. The abovementioned actions interrupted the standard educational curricula, and consequently possibly limited the early career surgeon in meeting mandatory requirements [5, 12].

After nearly 2 years, up to now in winter of 2021, there is still no normalization of the healthcare system in sight. The purpose of this study was to assess the effects of the SARS-CoV‑2 pandemic on the residency training for orthopedics and traumatology in Germany and to query residents’ expectations on their future education with an online based nationwide survey.

## Material and methods

### Study design and participants

A nationwide online-based cross-sectional anonymous voluntary study was conducted in order to query physicians within their residency training for orthopedics and traumatology in Germany. The survey was carried out between October 2021 and November 2021 and was distributed by the youth organization (“Junges Forum O und U”) of the German Society for Orthopedics and Traumatology (DGOU) and the German Professional Association for Orthopedics and Traumatology (BVOU) as well as by the youth organization (Forum AGA Assistenzärzte” of the Society for Arthroscopy and Joint Surgery (AGA) using their email lists. Overall, 789 resident physicians were contacted and asked to participate in the survey. Both youth organizations sent two reminders encouraging participation in the study to enhance the response rate.

### Questionnaire

The study was reviewed and approved by the hospital’s institutional ethics committee (#EA4/165/21) as an anonymous online study. All questions were developed by the first and senior author and were peer reviewed and approved by all co-authors. The study was provided as an online survey with the use of a commercially available product (SurveyMonkey Inc.; San Mateo, CA, USA; https://www.surveymonkey.com) and was designed in German language.

In total, the survey included 39 individual questions and statements with a total of 6 categories: A) general everyday clinical practice and care, B) surgery, C) continuing medical education, D) medical conferences, E) research, F) prospect on the future. Questions were grouped into four blocks. In block 1, closed questions (“applicable”, “not to answer”, “not applicable”) as well as percentage changes for certain questions (“-X%”, “0%”, “+X%”) were asked followed by block 2 and 3 which used a bipolar 5‑point-Likert scale. Here the possible responses were “totally agree” (corresponding to 1 on the scale), “rather agree” (0.75), “neutral” (0.50), “rather disagree” (0.25), “totally disagree” (0). Blocks 4–9 inquired about general information about the participant.

The full questionnaire, as well as an English translation can be found in the supplemental material (Supplement Survey DE, Supplement Survey ENG).

## Statistical analysis

Data analysis was performed using Excel 2019 Version 16.53 (Microsoft Corporation, Redmond, WA, USA). Descriptive statistical calculations were performed by calculating means and standard deviations (SDs) of the bipolar 5‑point Likert scales and the open questions.

## Results

A total of 103 responses were received representing a response rate of 13.1%. Eight participants had to be excluded because they had already finished their residency at the time of the survey. Therefore, 95 participants with an average age of 31.3 ± 2.8 years were included in the final analysis. Fifty-six (58.9%) participants were male. Overall, 40.0% were early career residents within the first 3 years of training and the remaining 60.0% were senior residents with 4 or more years of training. The minimum time for board certified completion of orthopedics and traumatology training in Germany comprises 6 years. When looking at the type of facility, the majority (75.8%) worked in a maximum care clinic or university hospital. Detailed basic information is summarized in Table 1.

**Table 1:** Basic information on participating residents

Variables	Total *n* = 95
*Gender*	
Female, *n* (%)	39 (41.1)
Male, *n* (%)	56 (58.9)
Mean age years (SD)	31.3 (2.8)
*Years in residency training*	
1, *n* (%)	5 (5.3)
2, *n* (%)	14 (14.7)
3, *n* (%)	19 (20.0)
4, *n* (%)	12 (12.6)
5, *n* (%)	21 (22.1)
6, *n* (%)	21 (22.1)
>6, *n* (%)	3 (3.2)
*Type of facility*	
Maximum care clinic/university hospital	72 (75.8)
Clinic with focused care	9 (9.5)
Basic and standard care clinic	12 (12.6)
Other	2 (2.1)

*SD* Standard deviation, *n* Number, *%* Percentage

### General everyday clinical practice and care

When asked whether the participating resident physicians were able to adequately discuss clinical decision regarding difficult outpatient and inpatient cases with their training supervisors despite the restrictions during the past year, 89.5% answered with “applicable”.

Regular morning meetings with the whole department including virtual ones took place in 38.9% and 61.1% denied this statement. Regular case discussions of patients awaiting surgery (indication discussion) took place in 34.1% and debriefing case discussions of the already operated patients in only 30.5%.

56.8% of all participants were furthermore assigned to non-specialty related care for COVID-19 patients with an average duration of 2.6 ± 1.8 months. Moreover, 68.4% stated that a split of staff for at least 1 month was in place in their department since the start of the pandemic. Here, at least one team of physicians was released from work in order to be able to step in within the working team in the event of an infection.

**Fig. 1:**
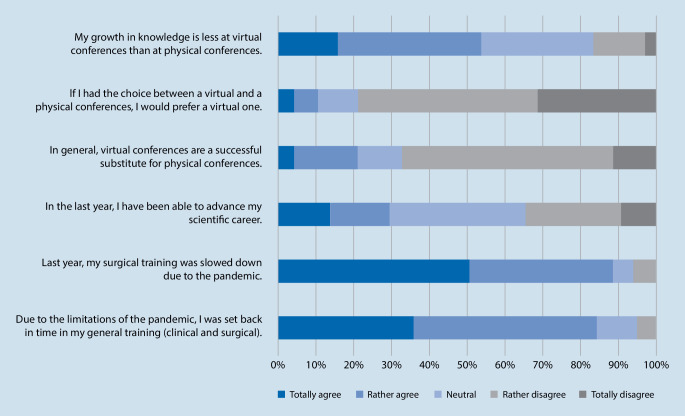
Results of the bipolar 5‑point Likert scale

Summarizing the general everyday clinical practice and care section, 80.0% of all residents fully or rather agreed with the statement that due to the limitations of the pandemic, they were set back in time of their general training (clinical and surgical) with a mean score of 0.8 ± 0.2 (Fig. 1).

### Surgery

When asked whether the resident has had the opportunity to actively participate in surgeries in the past year, 91.6% agreed (“applicable”) and only 7.4% disagreed (“not applicable”). However, the training in the OR was reduced by an average of 25.0 ± 25.3% in the last year, compared to prepandemic times, based on personal perception. Furthermore, 90.5% stated that in their institution experienced surgeons (specialist, attending, chief) operated more frequently due to time pressure.

In total 88.4% fully or rather agreed with the statement that in the past, their surgical training was slowed down due to the pandemic. Only 5.3% answered neutrally and 6.3% rather or completely disagreed (mean score 0.8 ± 0.2).

### Continuing medical education

Overall, 52.6% stated that residents were not able to finish their training because board examinations were suspended. 82.1% stated that there has been a change in number of institutional educational didactics (e.g. journal clubs, mortality and morbidity conferences) in their department compared to the previous year which resulted in an average reduction of 27.8 ± 30.9% institutional didactics. Furthermore, 90.5% stated that institutional courses were cancelled without an auxiliary date. Looking at external medical education courses such as Advanced Trauma Life Support (ATLS) or surgical courses, 67.4% indicated that they were not able to attend such courses due to the pandemic and for instance of being registered for such courses, they were cancelled without and auxiliary date in 65.3%.

### Medical conferences

On average 1.6 ± 1.0 conferences, the participating physicians planned to attend last year, were cancelled. Moreover, 76.8% answered “applicable” when being asked if a medical conference was cancelled without an auxiliary date.

91.6% of all participants took part in virtual medical conferences. However, only 21.1% fully or rather agreed that virtual conferences are a successful substitute for physical conferences and 67.4% rather or completely disagreed (mean score 0.4 ± 0.3). Moreover, when having the choice between a virtual and a physical conference, virtual conferences were highly undesirable (78.9% fully or rather agreed, mean score 0.3 ± 0.3). The majority (53.7%) fully or rather agreed that their growth in knowledge is less at virtual conferences compared to physical ones whereas 29.5% did not state a difference and 16.8% rather or completely disagreed (mean score 0.6 ± 0.3).

### Research

With regards to research opportunities, only 37.9% answered “applicable” and 49.5% “not applicable”, when being asked if the residents had the opportunity to invest time in research projects last year. Nevertheless, compared to pre-COVID-19 years, the average time invested in research slightly increased by 4.2% ± 23.2 but unfortunately at the same time 56.8% stated that they were involved in ongoing studies which were paused due to the pandemic. Asked if the participants were able to advance their scientific career, a mixed picture can be seen. 29.5% fully or rather agreed, 35.8% were neutral and 34.7% rather or completely disagreed (mean score 0.5 ± 0.3) Generally asked, whether there was a change in numbers of research projects that were newly developed last year compared to previous years within their institution, a heterogenic picture can be seen as well with 32.6% answering “applicable”, 28.4% “not applicable” or 38.9% “not to answer” each. In the 32.6%% with changes, the research activity on average increased by 7.1% ± 20.2.

### Prospect on the future

**Fig. 2:**
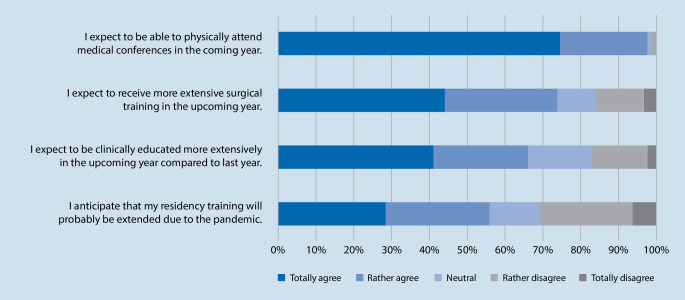
Prospect on the future results

Asking about the residents’ prospect on the future, 55.8% fully or rather agreed that their residency training will probably be extended due to the pandemic while 13.7% were neutral and only 30.5% rather or completely disagreed (mean score 0.6 ± 0.3). Nonetheless, 66.3% fully or rather agreed that they think they will clinically be educated more extensively in the upcoming year compared to last year while only 16.8% were neutral as well as rather or completely disagreed (mean score 0.7 ± 0.3). Regarding surgical training compared to last year, 73.7% fully or rather agreed that they expect to receive more extensive surgical training in the upcoming year (mean score 0.7 ± 0.3). Moreover, nearly everyone (97.9%) fully or rather agreed that they expect to be able to physically attend medical conferences in the coming year (Fig. 2).

## Discussion

This nationwide survey drastically shows how the SARS-CoV‑2 pandemic has impacted the residency training for orthopedics and traumatology in Germany after nearly 2 years.

In the everyday clinical practice and care, 80.0% of participants in our study claimed that they were set back in time of their general clinical training due to the pandemic. Despite the fact that nearly everyone (91.6%) could actively participate in surgeries, the OR time, however, has reduced on average by 25.0% compared to pre-pandemic times based on personal perception and 88.4% fully or rather agreed that within the last year, their surgical training was slowed down. Certainly, the reduced surgical experience is also based on the fact that overall fewer surgeries were allowed due to the restrictions.

In a survey focusing on the effects of COVID-19 on surgical training among resident physicians for general surgery from the USA by Aziz et al. [2] the phenomenon regarding OR experience can be seen as well. Here, a significant drop in number of cases performed by residents per week was displayed and 12.5% of junior residents were not allowed into the OR at all. Among surgical residents, fellows and early career surgeons Coleman et al. [5] were able to show that the majority of respondents (96%) reported that COVID-19 had a negative impact on their clinical experience. Furthermore, the study reported that 84% of residents experienced a greater than 50% reduction in operative volume [5]. Similar results can also be found for cardiothoracic surgical training with 75% of questioned residents reporting a reduction of time in the operation room and therefore expressing the immense disruption the pandemic created in surgical specialties [4, 24]. Less time in the operation theatre, however, can have intermediate negative effects for the next generation of orthopedic and trauma surgeons. With less elective cases due to pandemic-related restrictions and the overall restructuring of the healthcare sector, orthopedic and traumatology residents are experiencing a massive reduction in numbers of operations as seen above and breadth of pathologies they are exposed to. This may not only lead to decreased surgical skills but also to reduced discussion content for educational didactics [15].

Medical education for resident physicians was also affected heavily by the pandemic. Our study showed that on average 27.8% less institutional didactics were held and 67.4% of all participants indicated they were not able to attend external medical education courses such as ATLS courses which are an essential part of the residency training. The former is especially interesting because an enormous content of general didactic content has been developed since the beginning of the pandemic and as studies from the USA and UK showed, resident programs did conduct grand rounds and minor conferences using teleconferencing with an increase of attendance of resident physicians [2, 14, 16, 17, 24]. In numbers, the study by Aziz et al. [2] showed that 41.3% received more didactic teaching during the pandemic than in prior years. Additionally, studies also showed that webinars, online discussions, educational videos and simulations were popular among early career doctors in multiple specialties [6, 7, 9, 13, 27].

In contrast to the abovementioned didactics, this study showed that in total only 21.1% fully or rather agreed that virtual medical conferences were a successful substitute for physical conferences and the majority (53.7%) fully or rather agreed that their growth in knowledge is less participating in virtual conferences compared to physical ones. In line with our results, Rana et al. [19] showed that senior residents believed that virtual meetings were less effective than in-person meetings compared to junior residents. Despite the fact that online based didactics and educations formats are well-accepted, these findings show the desire of in person networking and face-to-face interactions. Thus, possible improvements of virtual formats need to be discussed.

Regarding research opportunities, compared to pre-COVID-19 years, the average time invested in research slightly increased to 4.2%. At the same time 56.8% of all participants stated that they were involved in ongoing studies which were paused during the pandemic. This is in harmony with the findings of Sohrabi et al. [22] showing that research, especially clinical research, has been especially challenging since the start of the pandemic.

By looking at the prospect on the future, 55.8% of the current study’s participants fully or rather agreed that their residency training will be extended due to the pandemic. Similar results can be found for other surgical specialties as well. Khalafallah et al. [14] showed a feeling of inability to meet Accreditation Council for Graduate Medical Education (ACGME) operative case minimums among neurosurgery residents by 67.6% and high concerns (65.8%) that COVID-19 hinders their surgical milestones. For general surgery, one survey revealed that 42.3% felt that they would not meet the ACGME case requirements and in a cardiothoracic surgery study, 71% raised their concerns that they might require an extension in their planned time of training [2, 4]. A study from the UK focusing on the impact of COVID-19 on orthopedic services and training also showed that 69% felt the pandemic would results in a delay of their training [15]. Despite these findings, the majority of our study population nonetheless has optimistic thoughts for the future and expect they will be educated more extensively both clinically and in the OR.

There are several limitations to our study. First, our overall response rate was 13% which may have underpowered our study. However, this response rate is comparable to other studies of this type. Second, there might have been selection bias as the survey was distributed through two big orthopedic and traumatology societies which not all residents are part of, however, these societies include the highest numbers of members for the forementioned specialty. Additionally, as especially residents from university hospitals are part of these societies, there might have been potential underreporting of our results for other (rural) facilities. Due to the specific question of this highly relevant topic, the survey has been developed by consensus. Due to these circumstances, a validation has not been performed, which must be considered a limitation. Nonetheless, with this study, resident physicians of all years were reached, and the findings may therefore be representative for orthopedic and traumatology residents across Germany.

## Conclusion

The SARS-CoV‑2 pandemic has significantly impacted the residency training for orthopedics and traumatology in Germany. In nearly all aspects resident physicians had to struggle with restrictions that negatively affected their training. Therefore, measures must be implemented in order to overcome possible leeway. For the future, we therefore strongly recommend to especially focus on training early career surgeons such as resident physicians and to give them the opportunity to participate in hands-on surgical course. This will not in total replace the lost experience caused the pandemic, but it will be a first step towards an improved education.

## Supplementary Information

ESM 1: Deutscher Fragebogen „Effects of the SARS-CoV‑2 Pandemic on the Residency Training in Orthopedics and Traumatology after nearly two years in Germany—A Nationwide Survey“ – Eine Umfrage des Centrums für Muskuloskeletale Chirurgie (CMSC) der Charité, Universitätsmedizin Berlin

ESM 2: English survey “Effects of the SARS-CoV‑2 Pandemic on the Residency Training in Orthopedics and Traumatology after nearly two years in Germany—A Nationwide Survey”—A survey of the Center for Musculoskeletal Surgery (CMSC) of the Charité, University Medicine Berlin

## Abbreviations

BVOU: German Professional Association for Orthopedics and Traumatology
COVID-19: coronavirus disease 2019
DGOU: German Society for Orthopedics and Traumatology
SARS-CoV‑2: severe acute respiratory syndrome coronavirus 2
WHO: World Health Organization

## Declarations

For this article no studies with human participants or animals were performed by any of the authors. All studies mentioned were in accordance with the ethical standards indicated in each case. IRB: Institutional review board approval was obtained. (IRB# EA4/165/21)
