# Supplementary material for: Effects of the SARS-CoV‑2 pandemic on residency training in orthopedics and traumatology in Germany: A nationwide survey
Source: Orthopadie (Heidelb). 2022 Aug 25;51(10):844–52. doi: 10.1007/s00132-022-04295-w (PMC9406266; doi:10.1007/s00132-022-04295-w)
Supplement: Supplementary file 1 — ESM 1: Deutscher Fragebogen „Effects of the SARS-CoV‑2 Pandemic on the Residency Training in Orthopedics and Traumatology after nearly two years in Germany—A Nationwide Survey“ – Eine Umfrage des Centrums für Muskuloskeletale Chirurgie (CMSC) der Charité, Universitätsmedizin Berlin [file 132_2022_4295_MOESM1_ESM.pdf]

Effects of the SARS-CoV-2 Pandemic on the Residency Training in Orthopedics and Traumatology after nearly two years in Germany – A Nationwide Survey

Eine Umfrage des Centrums für Muskuloskeletale Chirurgie (CMSC) der Charité – Universitätsmedizin Berlin

Dominik Adl Amini dominik.adl-amini@charite.de

Maximilian Müllner maximilian.muellner@charite.de

**I)**	**Welche Auswirkungen hatte die COVID-19 Pandemie auf Ihre Ausbildung im letzten Jahr?**	**Trifft zu**	**Nicht zu beant-worten**	**Trifft nicht zu**
**A)**	**Allgemeiner Klinikalltag/stationäre Versorgung:**			
1.	War es Ihnen im letzten Jahr trotz der Einschränkungen durch die Pandemie (z.B. Kontaktbeschränkungen) ausreichend möglich, mit Ihren Weiterbildungsverantwortlichen klinische Entscheidungen zu schwierigen ambulanten und stationären Patientenfällen zu besprechen?	☐	☐	☐
2.	Fanden in Ihrer Abteilung im letzten Jahr regelmäßige Frühbesprechungen mit der Möglichkeit der Teilnahme des kompletten Teams statt? (auch virtuell/online)	☐	☐	☐
3.	Fanden in Ihrer Abteilung im letzten Jahr regelmäßige Fallbesprechungen <u>der bereits operierten Patienten</u> mit der Möglichkeit der Teilnahme des kompletten verfügbaren Teams statt? (Nachbesprechung)	☐	☐	☐
4.	Fanden in Ihrer Abteilung im letzten Jahr regelmäßige Fallbesprechungen <u>der noch zu operierenden Patienten</u> mit der Möglichkeit der Teilnahme des kompletten verfügbaren Teams statt? (Indikationsbesprechung)	☐	☐	☐

5.	Wurden Sie „fachfremd“ in anderen Abteilungen/Stationen für die Versorgung von COVID-19 Patienten für eine Dauer von mindestens einem Monat eingesetzt?	☐	☐	☐
5.1	Falls zutreffend, wie viele Monate wurden Sie dort insgesamt eingesetzt?	Bitte Anzahl hier eintragen:		
6.	Gab es in Ihrer Abteilung im letzten Jahr eine Team-Teilung des Personals für mindestens einen Monat? (Jeweils ein von der Arbeit freigestelltes Team von Ärztinnen/Ärzten, welches im Falle einer Infektion innerhalb des arbeitenden Teams einspringen kann.)	☐	☐	☐
7.	Konnten in Ihrer Abteilung im letzten Jahr Ärztinnen/Ärzte in Weiterbildung diese pandemiebedingt nur verzögert beenden, da vorrübergehend keine Facharztprüfungen angeboten wurden?	☐	☐	☐
**B)**	**OP**			
1.	Operierten in Ihrer Abteilung im letzten Jahr aufgrund von Zeitdruck häufiger erfahrene Ärzte (Fachärztin/Arzt, Oberärztin/Arzt, Chefärztin/Arzt)?	☐	☐	☐
2.	Hatten Sie im letzten Jahr die Möglichkeit aktiv an Operationen teilzunehmen?	☐	☐	☐
3.	Um wieviel Prozent (%) hat sich Ihre Ausbildung im OP im letzten Jahr verändert? **(Bei weniger: „- X%“; bei mehr: „+ X%“; bei keiner Reduktion, bitte „0%“ angeben!)**	Bitte Anzahl hier eintragen:		

C)	**Fort- und Weiterbildungen**			
1.	Gab es in Ihrer Abteilung im letzten Jahr eine Änderung der Anzahl an **internen** Fort- und Weiterbildungen im Vergleich zu dem Vorjahr?	☐	☐	☐
1.1	Falls zutreffend, um wieviel Prozent haben sich diese **internen** Fort- und Weiterbildungen im letzten Jahr laut Ihrer Schätzung verändert? **(Bei weniger: „- X%"; bei mehr: „+ X%"; bei keiner Reduktion, bitte „0%" angeben!)**	Bitte Anzahl hier eintragen:		
1.2	Falls zutreffend, wieviel Prozent dieser **internen** Fort- und Weiterbildungen sind im letzten Jahr ohne Nachholtermin ausgefallen?	Bitte Anzahl hier eintragen:		
2.	Haben Sie im letzten Jahr geplante **externe** Fortbildungen und Kurse pandemiebedingt nicht besuchen können (z.B. AE-, ATLS-, AO- oder Röntgen-Kurse)?	☐	☐	☐
2.1	Falls zutreffend, wieviel Prozent dieser **externen** Fort- und Weiterbildungen sind im letzten Jahr ohne Nachholtermin ausgefallen?	Bitte Anzahl hier eintragen:		
D)	**Kongresse**			
1.	Haben Sie im letzten Jahr virtuell an Kongressen teilgenommen?	☐	☐	☐
2.	Wurden im letzten Jahr Kongresse, an denen Sie teilnehmen wollten (physisch oder virtuell), ohne Nachholtermin abgesagt?	☐	☐	☐
3.	Wie viele Kongresse, an denen Sie im letzten Jahr teilnehmen wollten, wurden abgesagt?	Bitte Anzahl hier eintragen:		
E)	**Wissenschaft**			
1.	Hatten Sie die Möglichkeit im letzten Jahr Zeit in Forschungsprojekte zu investieren?	☐	☐	☐
1.2	Falls zutreffend, wieviel Prozent Ihrer Zeit konnten Sie in Forschungsprojekte investieren? **(Bei weniger: „- X%"; bei mehr: „+ X%"; bei keiner Reduktion, bitte „0%" angeben!)**	Bitte Anzahl hier eintragen:		

2.	Gab es im letzten Jahr in Ihrer Abteilung im Vergleich zu den Vorjahren, eine Änderung der Anzahl von Forschungsprojekte/-vorhaben, die neu erarbeitet wurden?	☐	☐	☐
2.1	Falls zutreffend, um wieviel Prozent änderte sich die Anzahl von Forschungsprojekte/-vorhaben? **(Bei weniger: „- X%"; bei mehr: „+ X%"; bei keiner Reduktion, bitte „0%" angeben!)**	Bitte Anzahl hier eintragen:		
3.	Wurden im letzten Jahr pandemiebedingt bereits laufende Studien, an denen Sie mitarbeiten, pausiert?	☐	☐	☐

**II)**	**Wie bewerten Sie folgende Aussagen?**	**Stimme voll zu**	**Stimme eher zu**	**Neutral**	**Lehne eher ab**	**Lehne völlig ab**
**A)**	**Klinik/OP/Wissenschaft**					
1.	Durch die Einschränkungen der Pandemie wurde ich in meiner allgemeinen Ausbildung (klinisch und operativ) zeitlich zurückgeworfen.	☐	☐	☐	☐	☐
2.	Im letzten Jahr hat sich meine operative Ausbildung pandemiebedingt verzögert.	☐	☐	☐	☐	☐
3.	Im letzten Jahr konnte ich meine wissenschaftliche Karriere vorantreiben.	☐	☐	☐	☐	☐
**B)**	**Kongresse**					
1.	Generell betrachtet sind virtuelle Kongresse ein gelungener Ersatz von physischen Kongressen.	☐	☐	☐	☐	☐

2.	Wenn ich die Wahl zwischen einem virtuellen und einem physischen Kongress hätte, würde ich einen virtuellen bevorzugen.	☐	☐	☐	☐	☐
3.	Mein Wissenszuwachs ist bei virtuellen Kongressen geringer als bei physischen Kongressen.	☐	☐	☐	☐	☐

**III)**	**Geben Sie uns eine Einschätzung Ihres Ausblicks auf die Zukunft**	**Stimme voll zu**	**Stimme eher zu**	**Neutral**	**Lehne eher ab**	**Lehne völlig ab**
1.	Ich gehe davon aus, dass sich meine Facharztweiterbildung pandemiebedingt verlängern wird.	☐	☐	☐	☐	☐
2.	Ich gehe davon aus, dass ich im kommenden Jahr klinisch umfänglicher ausgebildet werden kann.	☐	☐	☐	☐	☐
3.	Ich gehe davon aus, dass ich im kommenden Jahr operativ umfänglicher ausgebildet werden kann.	☐	☐	☐	☐	☐
4.	Ich gehe davon aus, dass ich im kommenden Jahr physisch an Kongressen teilnehmen kann.	☐	☐	☐	☐	☐

IV)	Welches Geschlecht haben Sie?	
1.	Weiblich	☐
2.	Männlich	☐
3.	Divers	☐

V)	Wann haben Sie Geburtstag?
1.	Bitte Geburtsdatum eintragen. (TT/MM/JJ)

VI)	In welcher Position sind Sie aktuell tätig?	
1.	Assistenzärztin/Assistenzarzt bzw. Weiterbildungsassistentin/Weiterbildungsassistent in der Praxis	☐
2.	Fachärztin/Facharzt	☐

VII)	In welchem Ausbildungsjahr sind Sie aktuell? Bei Fachärztinnen/Fachärzten: In welchem Jahr nach dem Erwerb der Facharztqualifikation befinden Sie sich?	1	2	3	4	5	6	>6
		☐	☐	☐	☐	☐	☐	☐

VIII)	In welcher Art Einrichtung sind Sie aktuell (überwiegend) tätig? (eine Antwort)	
1.	Klinik der Grund- und Regelversorgung	☐
2.	Klinik mit Schwerpunktversorgung	☐
3.	Klinik der Maximalversorgung/Universitätsklinikum	☐
4.	Rehabilitationsklinik	☐
5.	Andere	☐
