# Supplementary material for: Effects of the SARS-CoV‑2 pandemic on residency training in orthopedics and traumatology in Germany: A nationwide survey
Source: Orthopadie (Heidelb). 2022 Aug 25;51(10):844–52. doi: 10.1007/s00132-022-04295-w (PMC9406266; doi:10.1007/s00132-022-04295-w)
Supplement: Supplementary file 2 — ESM 2: English survey “Effects of the SARS-CoV‑2 Pandemic on the Residency Training in Orthopedics and Traumatology after nearly two years in Germany—A Nationwide Survey”—A survey of the Center for Musculoskeletal Surgery (CMSC) of the Charité, University Medicine Berlin [file 132_2022_4295_MOESM2_ESM.pdf]

Effects of the SARS-CoV-2 Pandemic on the Residency Training in Orthopedics and Traumatology after nearly two years in Germany – A Nationwide Survey

A survey by the Center for Musculoskeletal Surgery (CMSC) at Charité – Universitätsmedizin Berlin

Dominik Adl Amini dominik.adl-amini@charite.de
Maximilian Muellner maximilian.muellner@charite.de

**I)**	**What impact did the COVID-19 pandemic have on your residency training last year?**	**Applicable**	**Not to answer**	**Not Applicable**
**A)**	**General everyday clinical practice and care:**			
1.	Despite the restrictions imposed by the pandemic (e.g., contact restrictions), were you able to adequately discuss clinical decisions regarding difficult outpatient and inpatient cases with your training supervisors during the past year?	☐	☐	☐
2.	Have there been regular morning meetings in your department in the last year with the possibility of participation of the whole team? (virtually or online)	☐	☐	☐
3.	Did your department have regular case discussions of the patients awaiting surgery with the possibility of participation of the whole team during the last year? (Indication discussion)	☐	☐	☐

4.	Did your department hold regular case discussions of patients already operated on in the last year with the possibility of participation of the whole team? (Debriefing)	☐	☐	☐
5.	Have you been assigned to "non-specialty" care for COVID-19 patients in other departments/ wards for a period of at least one month?	☐	☐	☐
5.1	If applicable, how many months in total you were assigned there?	Please enter quantity here:		
6.	Has your department had a team split of staff for at least one month in the last year? (In each case, a team of physicians released from work who can step in within the working team in the event of an infection).	☐	☐	☐
7.	Were residents in your department able to complete their training last year only after a delay due to the pandemic, as no specialist examinations were offered temporarily?	☐	☐	☐

**B)**	**OR**			
1.	Did experienced surgeons (specialist, senior physician, chief) operate more frequently in your department in the last year due to time pressure?	☐	☐	☐
2.	Have you had the opportunity to actively participate in surgeries in the past year?	☐	☐	☐
3.	By what percentage (%) has your training in the OR changed in the last year? **(If less: "- X%"; if more: "+ X%"; if no reduction, please specify "0%"!)**	Please enter quantity here:		
**C)**	**Continuing medical education:**			
1.	Has there has been a change in number of institutional educational didactics in your department in the last year compared to the previous year?	☐	☐	☐
1.1	If applicable, by what percentage do you estimate these internal didactics have changed in the last year? **(If less: "- X%"; if more: "+ X%"; if no reduction, please specify "0%"!)**	Please enter quantity here:		
1.2	If applicable, what percentage of these internal didactics were cancelled without an auxiliary date last year? **(If less: "- X%"; if more: "+ X%"; if no reduction, please specify "0%"!)**	Please enter quantity here:		

2.	Have you been unable to attend planned external medical education courses in the past year due to the pandemic (e.g., AE, ATLS, AO, or radiology courses)?	☐	☐	☐
2.1	If applicable, what percentage of these external medical education courses were cancelled last year without an auxiliary date?	Please enter quantity here:		
**D)**	**Medial conferences**			
1.	Have you attended conferences virtually in the last year?	☐	☐	☐
2.	In the past year, have any conferences you wanted to attend (physical or virtual) been canceled without an auxiliary date?	☐	☐	☐
3.	How many conferences were cancelled you planned to attend last year?	Please enter quantity here:		
**E)**	**Research**			
1.	Did you have the opportunity to invest time in research projects last year?	☐	☐	☐
1.2	If applicable, what percentage of your time could you invest in research projects? **(If less: "- X%"; if more: "+ X%"; if no reduction, please indicate "0%"!)**	Please enter quantity here:		

2.	Was there a change in the number of research projects that were newly developed in your department last year compared to previous years?	☐	☐	☐
2.1	If applicable, by what percentage did the number of research projects change? **(If less: "- X%"; if more: "+ X%"; if no reduction, please specify "0%"!)**	Please enter quantity here:		
3.	Were any ongoing studies that you were involved in paused last year due to the pandemic?	☐	☐	☐

**II)**	**How do you rate the following statements?**	**Totally agree**	**Rather agree**	**Neutral**	**Rather disagree**	**Totally disagree**
**A)**	**Clinic/Surgery/Research**					
1.	Due to the limitations of the pandemic, I was set back in time in my general training (clinical and surgical).	☐	☐	☐	☐	☐
2.	Last year, my surgical training was slowed down due to the pandemic.	☐	☐	☐	☐	☐
3.	In the last year, I have been able to advance my scientific career.	☐	☐	☐	☐	☐

B)	Medial conferences					
1.	In general, virtual conferences are a successful substitute for physical conferences.	☐	☐	☐	☐	☐
2.	If I had the choice between a virtual and a physical conferences, I would prefer a virtual one.	☐	☐	☐	☐	☐
3.	My growth in knowledge is less at virtual conferences than at physical conferences.	☐	☐	☐	☐	☐

III)	Give us a prospect on your future	Totally agree	Rather agree	Neutral	Rather disagree	Totally disagree
1.	I anticipate that my residency training will probably be extended due to the pandemic.	☐	☐	☐	☐	☐
2.	I expect to be clinically educated more extensively in the upcoming year compared to last year.	☐	☐	☐	☐	☐
3.	I expect to receive more extensive surgical training in the upcoming year.	☐	☐	☐	☐	☐

4.	I expect to be able to physically attend medical conferences in the coming year.	☐	☐	☐	☐	☐

IV)	**Gender**	
1.	female	☐
2.	male	☐
3.	diverse	☐

V)	**Date of birth**
1.	Please enter date of birth. (DD/MM/YY)

VI)	**What is your current position?**	
1.	Resident physician	☐
2.	Specialist	☐

VII)	**In which year of your residency training are you currently? For specialists: In which year after board certification are you?**	**1**	**2**	**3**	**4**	**5**	**6**	**>6**
		☐	☐	☐	☐	☐	☐	☐

VIII)	In what type of facility are you currently (predominantly) employed? (one answer)	
1.	Basic and standard care clinic	☐
2.	Clinic with focused care	☐
3.	Maximum care clinic/university hospital	☐
4.	Rehabilitation clinic	☐
5.	Other	☐
